# Maternal type 1 diabetes, pre-term birth and risk of autism spectrum disorder–a prospective cohort study

**DOI:** 10.1093/ije/dyac116

**Published:** 2022-06-03

**Authors:** Martina Persson, Abraham Reichenberg, Mikael Andersson Franko, Sven Sandin

**Affiliations:** Department of Clinical Science and Education, Division of Pediatrics, Karolinska Institutet, Stockholm, Sweden; Department of Psychiatry, Icahn School of Medicine at Mount Sinai, New York, USA; Seaver Autism Center for Research and Treatment at Mount Sinai, New York, USA; Department of Psychiatry, Icahn School of Medicine at Mount Sinai, New York, USA; Seaver Autism Center for Research and Treatment at Mount Sinai, New York, USA; Department of Clinical Science and Education, Division of Pediatrics, Karolinska Institutet, Stockholm, Sweden; Department of Medical Epidemiology and Biostatistics, Karolinska Institutet, Stockholm, Sweden; Department of Psychiatry, Icahn School of Medicine at Mount Sinai, New York, USA; Seaver Autism Center for Research and Treatment at Mount Sinai, New York, USA; Department of Medical Epidemiology and Biostatistics, Karolinska Institutet, Stockholm, Sweden

**Keywords:** Autism, type 1 diabetes, pre-term birth, epidemiology, mediation, cohort, epidemiology, aetiology

## Abstract

**Background:**

It has been suggested that maternal type 1 diabetes (T1D) increases the risk of autism spectrum disorder (ASD) in the offspring. However, it is unclear whether this risk is mediated by pre-term birth, affecting around one-third of pregnancies with T1D, and whether maternal levels of glycated haemoglobin (HbA1c) impact the risk.

**Methods:**

A cohort of 1.4 million Swedish children born between 1998 and 2015, and their parents. Maternal T1D and HbA1c before or in early pregnancy, gestational and ASD diagnoses were obtained from Swedish national registers. Relative risk (RR) and 95% CIs of ASD were estimated by hazard ratios (HRs) from Cox regression or RR from log-binomial regression.

**Results:**

Of 1 406 650 children, 8003 (0.6%) were born to mothers with T1D, 24 941 (1.8%) were diagnosed with ASD and 81 915 (5.8%) were born pre-term. The risk of ASD was increased in offspring of mothers with T1D was HR = 1.40 (1.21–1.61). The RR for each +5-mmol/mol excess HbA1c was estimated at HR = 1.03 (0.97–1.10). The T1D effect on ASD mediated through pre-term birth was estimated at RR = 1.06 (1.05 to 1.08), corresponding to 22% (16% to 41%) of the total effect. T1D in pregnancy was associated with increased ASD risk in the offspring. Twenty percent of the total effect was accounted for by pre-term birth. HbA1c was not associated with ASD risk, beyond the risk associated by the T1D diagnosis itself.

**Conclusion:**

Awareness of ASD in the offspring of mothers with T1D may be warranted, especially considering the additional effect of pre-term birth.

Key Messages

- In this cohort study of 1.4 million Swedish children and close to 30 000 autism cases, we found a 1.4-fold increased risk of autism in the offspring of mothers with type 1 diabetes diagnosed before child birth.
- We did not find any support for the notion that increasing glycated haemoglobin (HbA1c) levels before or during early pregnancy would increase the risk further.
- Approximately 20% of the total risk of autism spectrum disorders in the offspring was mediated through the pathway of pre-term birth.

## Introduction

Type 1 diabetes (T1D) in pregnancy increases risks of pre-term birth^1^ and severe fetal and neonatal complications.^2^ However, data are limited on long-term neurological outcomes in offspring of mothers with T1D.

Autism spectrum disorder (ASD) is characterized by impairment in social communication and repetitive and restricted behaviours.^3^ ASD affects 1–2% of children worldwide.^4^^,^^5^ The contribution of genetic variance is substantial^6^^,^^7^ but a number of environmental exposures have also been associated with increased risk.^8^

Studies on the association between maternal diabetes and ASD reported mixed results.^9–16^ Meta-analyses, with focus on pre-gestational diabetes, support an overall association.^17^^,^^18^ However, existing individual studies had limited sample size, did not distinguish between different types of diabetes and included varying definitions of ASD limiting generalizability. Also, previous studies did not address the potential role of pre-term birth. Women with T1D often give birth at older ages and are at increased risk of pre-term delivery.^1^ Both pre-term birth and higher maternal age are associated with ASD risk^8^ and maternal age is an indicator of T1D duration. Also, level of glycaemic control in mothers with T1D has been suggested to modify ASD risk.^19^^,^^20^

The study objective is to test the hypothesis that maternal T1D increases the risk of ASD in the offspring. We controlled for important confounding, examined the potential modifying role of pre-term birth and investigated whether ASD risk can be linked to maternal blood glucose levels, measured as glycated haemoglobin (HbA1c).

## Methods

### Study population

The study population included all children born alive in Sweden between 1998 and 2015 to mothers from the Nordic countries (Sweden, Denmark, Finland, Iceland or Norway) as recorded in the Swedish Medical Birth register (MBR). MBR records data on all pregnancies and deliveries in Sweden since 1973, with almost complete coverage. In Sweden, healthcare is free of charge and accessible to all citizens, which minimizes the risk of selection bias. Individual-level information from the Swedish national registries is linked by the unique personal identification numbers of all citizens.^21^

In Sweden, all infants and pre-school children regularly undergo routine medical and developmental examinations. At age 4 years, a developmental assessment (motor skills, language, cognitive and social development) is performed. Children with suspected developmental disorders are referred for further investigation by a specialized team at a child psychiatry unit or habilitation service. Diagnostic information is reported to the Swedish National Patient Register (NPR). The NPR includes all psychiatric inpatient diagnoses in Sweden since 1973, somatic diseases since 1987 and outpatient visits from 2001. The diagnoses in the NPR are assigned by clinical specialists, using, since 1997, the 10th version of the International Coding of Diseases (ICD 10). NPR has been subject to extensive validation efforts.^21^ ASD and T1D codes are presented in Supplementary Table S1 (available as Supplementary data at *IJE* online).

## Covariates

Women with T1D were identified in the NPR and the MBR. Data on maternal age at delivery, body mass index (BMI; kg/m^2^) at first antenatal visit, gestational age (weeks) and offspring sex were retrieved from the MBR. Gestational age is estimated at the early second-trimester ultrasound examination or, in <1% of births, using the date of last menstrual period. In Sweden, patients with T1D are cared for at university clinics by specialists in diabetes/endocrinology. Diagnosis of T1D is based on criteria according to the International Society for Pediatric and Adolescent Diabetes^22^ and the American Diabetes Association.^23^ In early pregnancy, women with T1D are referred to specialist antenatal care and supervised by a team of obstetricians, midwifes, diabetes nurses and diabetologists.

Data on glycated haemoglobin (HbA1c mmol/mol) were obtained from the National Diabetes Register (NDR) and recordings within –365 to +90 days from estimated conception were used in the analyses. HbA1c reflects the average level of blood glucose over the last 6–8 weeks and according current guidelines HbA1c should ideally be kept at <48 mmol/mol in early pregnancy.^24^ NDR started operating in 2001 and covers a majority of individuals with T1D in Sweden.^25^ Paternal age is correlated with maternal age, has been associated with both pre-term birth and ASD, and can therefore introduce confounding.^26^^,^^27^ Data on paternal age were obtained from the Swedish Multi-generational Register. BMI (kg/m^2^) at the first antenatal visit was calculated based on self-reported height and measured weight, and categorized as underweight: BMI < 18.5 kg/m^2^, normal weight: BMI 18.5–24.9, overweight BMI: 25–29.9 and obesity BMI > 30.^28^

Maternal and paternal socio-economic status (SES) affects overall health and the degree of health-seeking behaviour, and was considered as a potential confounder.^29^ We estimated SES by maximum attained education at date of delivery, based on information from Statistics Sweden, the Swedish government body for official statistics. Level of education was categorized as ‘primary’, i.e. elementary school; ‘secondary’, i.e. upper secondary school; and ‘university’, i.e. university degree. Psychiatric disease is more common in T1D^30^ and in individuals with ASD,^31^ and may therefore confound the association between T1D and ASD. Thus, parental psychiatric history was included as a potential confounder.

### Statistical methods

Relative risk (RR) of ASD was estimated by hazard ratios (HRs) and associated two-sided 95% Wald CIs calculated from Cox proportional hazards regression models. Each child was followed for incident ASD diagnosis from 1 year of age until the first ASD diagnosis, emigration, death or end of follow-up on 31 December 2017, whichever came first. First, we fitted a ‘crude model’, examining risks associated with maternal T1D adjusted for birth year as a continuous variable. In a second model, we adjusted for potential confounding by additionally including covariates for maternal and paternal age, history of maternal and paternal psychiatric disorders at delivery (yes/no) and maternal and paternal years of education at delivery. Birth year and parental ages entered the models using natural cubic splines with 3 degrees of freedom, except for the analyses stratified by T1D in which the small number of observations only allowed 2 degrees of freedom.

#### Mediation by pre-term birth

Since mothers with T1D have an increased risk of pre-term birth^2^ and pre-term birth is associated with increased risk of ASD,^32^ we examined the modifying role of pre-term birth (Supplementary Figure S7, available as Supplementary data at *IJE* online). First, we repeated the analyses in subgroups of children born term/pre-term and in singletons since twins are more often born pre-term and at increased risk of ASD.^33^ Next, we performed a mediation analysis of pre-term birth by approximating our Cox models with log-binomial regression and fitting Natural Effects Models.^34^^,^^35^ These analyses are using two log-binomial regression models, one outcome model including T1D as a predictor for offspring ASD risk and one mediation model including T1D as a predictor for pre-term birth. We adjusted for confounding as above, and risks were estimated separately for male and female offspring. Additionally, we analysed how much of the mediation was attributed to interaction between T1D and pre-term birth. We calculated E-values for the direct and mediated effects.^36^ The E-value for the direct effect estimates the effect size of an unmeasured confounder associated with both T1D and ASD required to completely explain away an estimated direct effect, and similar for the mediated effect (associated with both T1D and pre-term birth). We calculated percentile based 95% bootstrap CIs.^37^ Computer code for the mediation analysis is shown in Supplementary Table S10 (available as Supplementary data at *IJE* online).

##### Glucose levels—HbA1c

We extended the crude Cox regression model with HbA1c (mmol/mol) data and examined ASD risk associated with HbA1c, above what can be attributed to a T1D diagnosis alone. This was achieved by a parametrization in which we added a continuous variable taking the value zero for women without T1D, and HbA1c minus the average HbA1c for women with T1D for women with T1D. This two-stage risk model allowed us to estimate the risk associated with a T1D diagnosis, as well as the additional excess risk associated with a change in HbA1c in the average T1D mother by fitting a natural cubic spline to the HbA1c data. We compared the distribution of study covariates between mothers with T1D with and without data on HbA1c.

#### Supplementary analyses

We performed a series of complementary analyses; (i) since obesity is a growing problem in pregnant women with T1D and may increase insulin resistance and hyperglycemia, we examined ASD risk in subgroups by maternal BMI; (ii) ASD risk in relation to maternal T1D was also compared in subgroups of small-, appropriate- and large-for-gestational-age offspring; (iii) due to the skewed sex ratio in ASD, we examined risks of ASD in male and female offspring separately and in term and pre-terms; (iv) since T1D duration is closely linked to maternal age^38^ and T1D may affect biological age,^39^ we estimated the risk of offspring ASD as a function of maternal age for mothers with and without T1D; (v) the analyses were re-run for the subset of individuals with autistic disorder (AD), a classification present in the ICD-10 coding system for the most severely affected individuals.

All analyses were performed using the statistical package R, version 3.6.1 (function coxph in package survival) and SAS 9.4 (proc causalmed). Computer code is available from the corresponding author by request. We examined the assumption of proportional hazards by visual inspection of weighted Schoenfeld residuals and score tests.^40^ All tests of statistical hypotheses were done on the two-sided 5% level of significance corresponding to two-sided 95% CIs covering the value one. We did not adjust for multiplicity of statistical tests; however, the primary hypothesis is addressed using a single test. No imputation was done for missing data on covariates.

## Results

The study cohort included 1 421 082 children. We excluded 40 children diagnosed with ASD before age 1 year. Only 14 392 (1.0%) did not have complete data on all covariates and were excluded from the statistical analyses. Thus, our analyses included 1 406 650 children, of whom 8003 (0.6%) children were born to mothers with T1D. There were 196 (2.4%) children diagnosed with ASD to mothers with T1D compared with 24 745 (1.8%) in children of mothers without T1D corresponding to 275 cases per 100 000 person-years among offspring of mothers and 184 cases per 100 000 person-years among mothers without T1D. The median length of follow-up was 8.5 person-years for offspring of mothers without T1D and 9.5 person-years for offspring of mothers with T1D (Table 1; Supplementary Figure S1, available as Supplementary data at *IJE* online).

**Table 1:** Cohort description

Covariate	Mothers without T1D [number of children (%)]	Mothers with T1D [number of children (%)]
Children (% male)	13 98 647 (51.5%)	8 003 (51.0%)
Person-years/length of follow-up [median (min, max)]	8.5 (0.0, 19.0)	9.5 (0.1, 19.0)
Autism spectrum disorder (ASD)	24 745 (1.8%)	196 (2.4%)
Incidence rate of ASD per 100 000 person-years (total follow-up)	184 (13 436 700)	275 (71 147)
Autistic disorder (AD)	13 277 (0.95%)	103 (1.29%)
Incidence rate of AD per 100 000 person-years (total follow-up)	99 (13 484 812)	145 (71 535)
Pre-terms (<37 weeks’ gestation)	80 056 (5.7%)	1 859 (23.2%)
Birth year		
1998–2003	432 447 (30.9%)	2 029 (25.3%)
2004–2009	480 012 (34.3%)	2 771 (34.6%)
2010–2015	486 188 (34.8%)	3 203 (40.0%)
Mother’s age at delivery (years)		
<20	11 890 (0.9%)	73 (0.9%)
20–29	536 402 (38.4%)	3166 (39.6%)
30–39	789 826 (56.5%)	4 402 (55.0%)
≥40	60 529 (4.3%)	362 (4.5%)
Father’s age at delivery (years)		
<20	3 851 (0.3%)	18 (0.2%)
20–29	357 380 (25.6%)	2 079 (26.0%)
30–39	855 114 (61.1%)	4 831 (60.4%)
≥40	182 302 (13.0%)	1 075 (13.4%)
Size for gestational age^b^		
SGA	25 817 (1.9%)	92 (1.2%)
AGA	1 278 274 (94.3%)	4 771 (61.4%)
LGA	50 867 (3.8%)	2 908 (37.4%)
Maternal psychiatric history	130 821 (9.4%)	1 346 (16.8%)
Paternal psychiatric history	83 071 (5.9%)	1 588 (7.3%)
Mother's BMI (Q1/Median/Q3)^a^^,b^	23.5 (21.5–26.5)	25.1 (22.8–28.3)
Underweight^a^	26 908 (2.1%)	35 (0.5%)
Normal weight^a^	785 401 (62.2%)	3 537 (49.0%)
Overweight^a^	305 570 (24.2%)	2 410 (33.4%)
Obese^a^	144 031 (11.4%)	1 237 (17.1%)
Maternal education		
Primary	113 469 (8.1%)	784 (9.8%)
Secondary	614 441 (43.9%)	3 716 (46.4%)
University	670 737 (48.0%)	3 503 (43.8%)
Paternal education		
Primary	144 600 (10.3%)	892 (11.1%)
Secondary	715 042 (51.1%)	4 406 (55.1%)
University	539 005 (38.5%)	2 705 (33.8%)

T1D, type 1 diabetes; BMI, body mass index; SGA, small for gestational age; AGA, appropriate for gestational age; LGA, large for gestational age.

a Underweight: BMI < 18.5, normal weight: BMI 18.5–25, overweight: BMI 25–30, obese: BMI ≥ 30.

b Q1: 1st quartile (25th percentile), Q3: 3rd quartile (75th percentile); 140 225 (9.8%) missing values for BMI and 45 058 (3.2%) for size for gestational age were excluded.

The risk of ASD was increased in offspring of mothers with T1D. In the crude model, only adjusted for birth year, the HR for ASD in offspring of mothers with T1D compared with offspring of mothers without T1D was estimated as 1.52 (95% CI: 1.32–1.75). After adjustment for confounding, the HR for ASD was estimated at 1.40 (1.21–1.61), which remained robust when excluding twins (Table 2). There was no evidence for non-proportional hazard (Supplementary Figure S3, available as Supplementary data at *IJE* online).

**Table 2:** Risk of autism spectrum disorder in offspring of mothers with type 1 diabetes, compared with offspring of mothers without type 1 diabetes

Model	Number of patients	Rate (cases; person-years)	Hazard ratio (95% CI)
Crude	1 406 650	185 (24 941; 13 507 847)	1.52 (1.32–1.75)
Model 1 (adjusted)^a^			1.40 (1.21–1.61)
Subgroup analyses: Model 1—Singletons^b^	1 365 518	185 (24 205; 13 103 299)	1.41 (1.23–1.63)

Crude: adjusting for birth years by natural cubic splines.

a Model 1: Crude + adjusted for maternal and paternal age by natural cubic splines, parental psychiatric history (yes/no) and maternal and paternal education at delivery;

b Model 1 excluding twins, i.e. singletons only.

### Mediation by pre-term birth

In pregnancies without T1D, 81 915 (5.8%) of the offspring were born pre-term (Table 1) compared with 1859 (23.2%) of pregnancies to T1D mothers (Table 1). Among term-born children, the adjusted HR for ASD was 1.35 (1.14–1.60) and it was 1.20 (0.92–1.56) for pre-term-born children. There were only minor differences when restricting to singletons (Table 3; Supplementary Figure S2, available as Supplementary data at *IJE* online).

**Table 3:** Hazard ratios for autism spectrum disorder in subgroups of term and pre-term births

Model	Pre-term-born children			Term-born children		
	Number of patients	Rate (cases; person-years)	Hazard ratio (95% CI)	Number of patients	Rate (cases; person-years)	Hazard ratio (95% CI)
Crude	81 915	265 (2 109; 796 499)	1.32 (1.01–1.71)	1 324 735	180 (22 832; 12 711 348)	1.45 (1.23–1.72)
Adjusted^a^			1.20 (0.92–1.56)			1.35 (1.14–1.60)
Adjusted, singletons^b^	64 163	282 (1 751; 621 198)	1.17 (0.89–1.53)	1 301 355	180 (22 454; 12 482 101)	1.37 (1.16–1.62)

CI, two-sided 95% confidence interval.

a Adjusted for birth year, parental age by natural splines, parental psychiatric history and education.

b Adjusted model in subset including singletons only. Rate: cases per 100 000 person-years.

The mediation analysis showed that the direct risk of ASD from T1D was estimated at RR = 1.29 (1.11–1.48) and the risk mediated by pre-term birth was estimated at RR = 1.06 (1.05–1.08), unaffected by including an interaction term between T1D and pre-term birth (Supplementary Table S9, available as Supplementary data at *IJE* online) and corresponding to 22% (16–39%) of the total effect. In subgroups by sex, the percentage mediation from pre-term birth was estimated at 24% (15–73%) for males and 18% (12–33%) for females (Table 4). As measured by the E-values, an unmeasured confounder associated with both T1D and ASD with an approximate RR of ≥1.90 for both would be needed to explain away the direct effect. For the observed mediated effect, an approximate RR of 1.33 for both pre-term birth and ASD would suffice (Table 4).

**Table 4:** Mediation of effect from maternal type 1 diabetes to offspring autism spectrum disorder, mediated by pre-term birth

Effect estimates in the mediation analyses	Full data set of 1 406 650 patients, 24 941 with ASD (1.77%)		Subset male offspring		Subset female offspring	
	Crude model RR (95% CI)^a^	Adjusted model RR (95% CI)^b^	Crude model RR (95% CI)^a^	Adjusted model RR (95% CI)^b^	Crude model RR(95% CI)^a^	Adjusted model RR (95% CI)^b^
Total effect	1.51 (1.31–1.73)	1.37 (1.18–1.57)	1.41 (1.20–1.66)	1.29 (1.07–1.52)	1.80 (1.38–2.24)	1.61 (1.26–2.04)
E-value	2.39 (1.95–2.86)	2.09 (1.64–2.51)	2.16 (1.69–2.71)	1.90 (1.34–2.41)	2.99 (2.11–3.90)	2.59 (1.84–3.51)
NDE	1.40 (1.22–1.61)	1.29 (1.11–1.48)	1.31 (1.12–1.55)	1.22 (1.01–1.44)	1.66 (1.28–2.06)	1.50 (1.17–1.91)
Percentage	79 (69–84)	78 (62–84)	77 (57–85)	76 (27–85)	82 (72–88)	82 (67–88)
E-value	2.15 (1.73–2.61)	1.90 (1.47–2.32)	1.96 (1.48–2.48)	1.74 (1.13–2.23)	2.70 (1.88–3.55)	2.37 (1.62–3.23)
NIE	1.08 (1.07–1.09)	1.06 (1.05–1.08)	1.07 (1.06–1.08)	1.06 (1.05–1.07)	1.08 (1.07–1.11)	1.07 (1.05–1.09)
Percentage	21 (16–31)	22 (16–39)	23 (15–43)	24 (15–73)	18 (12–28)	18 (12–33)
E-value	1.37 (1.33–1.40)	1.33 (1.29–1.36)	1.34 (1.30–1.38)	1.31 (1.27–1.34)	1.39 (1.33–1.46)	1.35 (1.29–1.41)

Mediation analyses assessing risk by relative risk (RR) from log-binomial regression T1D → ASD and for pre-term → ASD.

ASD, autism spectrum disorder; T1D, type 1 diabetes; RR, relative risk from log-binomial regression; CI, two-sided confidence interval from bootstrapping 1000 samples.

a Outcome and mediation model adjusted for birth year (1998–2002, 2003–2007, 2008–2012, 2013–2015).

b Outcome and mediation model additionally adjusted for maternal age (<20, 20–24, 24–29, 30–34, 34–39, 40–44, >45), paternal age (<20, 20–24, 24–29, 30–34, 34–39, 40–44, >45), maternal psychiatric history at delivery (yes/no), paternal psychiatric history at delivery (yes/no), maternal education attainment at delivery (‘Grundskola’, ‘Gymnasium’, ‘University’) and paternal education attainment at delivery (‘Grundskola’, ‘Gymnasium’, ‘University’).

Total effect = NDE + NIE. ‘Natural direct effect’ (NDE) is the RR of ASD comparing offspring of mothers with T1D diagnosis with offspring of mothers without T1D diagnosis, when the pre-term covariates are (contrafactually) assigned the same value, e.g. term. ‘Natural indirect effect’ (NIE) is the RR of ASD comparing offspring born pre-term to offspring born term assuming all are born to mothers diagnosed with T1D. ‘Total effect’ is the RR of ASD comparing pre-term-born offspring of mothers with T1D diagnosis to term-born offspring of mothers without T1D diagnosis, i.e. the RR comparing assumed highest risk group to lowest risk group.

### Glucose levels—HbA1c

HbA1c measured 1 year prior to and until + 90 days from conception was available for 4945 (62%) of the mothers with T1D (Supplementary Figure S6, available as Supplementary data at *IJE* online) and distribution at the 10th percentile at 40, median at 58 and the 95th percentile at 85 mmol/mmol. There were no differences in the distribution of confounding factors for T1D mothers with and without data on HbA1c (Supplementary Table S2, available as Supplementary data at *IJE* online). The HR of ASD in the offspring of women with T1D with data on HbA1c compared with the offspring of women T1D was estimated at HR = 1.62 (1.34–1.97), similar to the HR for T1D in the main analysis.

**Figure 1:**
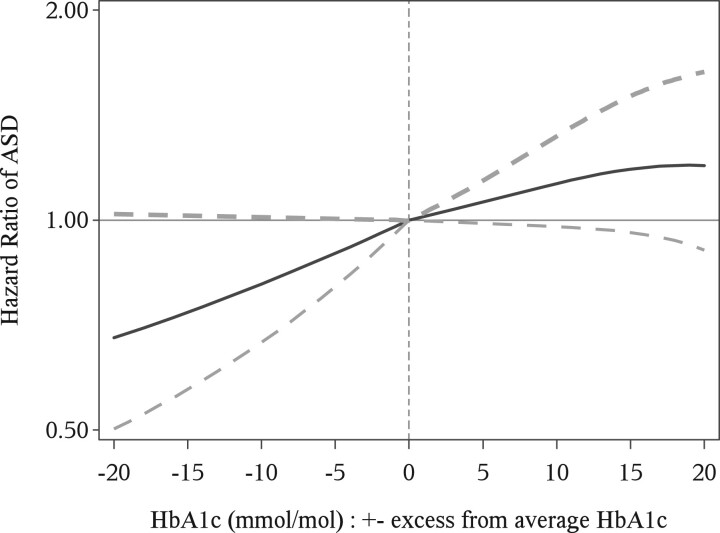
Hazard ratio of autism spectrum disorder (ASD) and two-sided 95% point-wise confidence intervals, associated with –20 to +20 deviation of maternal glycated haemoglobin (HbA1c) from average HbA1c in mothers with type 1 diabetes: excess risk on top of the risk associated with type 1 diagnosis alone The average HbA1c value was 58 mmol/mol.

The two-stage analysis, estimating the impact of HbA1c adding on to the risk of T1D diagnosis alone, showed an approximately linear form (Figure 1). The excess risk associated with a +5-mmol/mol excess in HbA1c was estimated at 1.03 (0.97–1.10) compared with offspring of mothers with T1D and average HbA1c.

### Supplementary analyses

There were no differences in ASD risk by maternal BMI (Supplementary Table S3, available as Supplementary data at *IJE* online) or offspring size for gestational age (Supplementary Table S4, available as Supplementary data at *IJE* online). The HRs in the earliest and latest birth cohorts were similar (Supplementary Table S5, available as Supplementary data at *IJE* online). Sex-specific analyses showed slightly higher HR point estimates for ASD in girls than in boys (Supplementary Tables S6 and S7 and Supplementary Figure S4, available as Supplementary data at *IJE* online). The risk of ASD associated with advancing maternal age was more pronounced, i.e. steeper, for offspring of mothers with T1D compared with offspring of mothers without T1D (Supplementary Figure S5, available as Supplementary data at *IJE* online). For AD, the direct risk of AD from T1D was estimated at RR = 1.19 (0.98–1.46) and the risk mediated by pre-term birth was estimated at RR = 1.09 (1.07–1.10) corresponding to 35% (20–100%) of the total effect. The E-values for the mediated effect indicate that a RR of 1.33 or higher for both pre-term birth and ASD would suffice to explain away the effect mediated through pre-term birth (Supplementary Table S8, available as Supplementary data at *IJE* online).

## Discussion

In a nationwide, prospective cohort study of 1.4 million live births in Sweden we found an increased risk of ASD in offspring of mothers with T1D. The results remained after a detailed adjustment for individual and familial confounding. A mediation analysis of pre-term birth indicated that ∼22% of the ASD risk was mediated through pre-term birth. The level of HbA1c before or in early pregnancy did not provide any additional risk modification.

Strengths of our study include the large, prospectively collected, nationwide sample with diagnostic ascertainment of T1D and ASD by clinical specialists. The inclusion of entire birth cohorts, with essentially complete follow-up through national health registries, minimizes risk of selection bias. Our detailed data enabled mediation analyses of pre-term birth in the association between T1D and risk of ASD, a detailed adjustment for confounding and to examine the association between HbA1c, an established disease biomarker and ASD risk. In all epidemiological studies, RR estimates rely on assumptions of no unmeasured confounding. Mediation analyses, as done here, additionally rely on assumptions of no, or low, unmeasured confounding between T1D and pre-term birth, T1D and ASD, and between pre-term birth and ASD. A mediation effect of 22% of the total risk may not seem small considering that causes of pre-term birth are heterogeneous and might not represent a single biological mechanism, and the confidence interval does not preclude an effect as high as 41%. Still, the calculated E-values were 1.9 for the direct T1D effect and 1.3 for the mediated effect. Besides the covariates we included in our models, the current scientific literature, as far as we know, does not suggest any additional potential confounders with RRs for ASD and T1D of >1.9. Known risk factors for T1D are mainly genetic and pleiotropic genes for T1D and ASD with RR of >1.9 have not yet been found.^41^^,^^42^ For the mediated effect, a currently unknown risk factor of RR of ≥1.3 confounding the relation between T1D, pre-term birth (primarily of environmental origins) and ASD cannot be ruled out, e.g. due to comorbid conditions such as pre-eclampsia and hypertension. It is also possible that oxidative stress and inflammation induced by hyperglycemia contribute to both pre-term birth and ASD risk.^8^ Still, an association with both ASD and pre-term birth with RR = 1.3 is fairly high considering currently known risk factors.^42^

Study limitations include lack of information on phenotype characteristics of the affected individuals, such as IQ score. However, the mediation effect through pre-term birth was higher with non-overlapping CIs when analyses were restricted to AD—a more severe form of ASD with higher rates of intellectual disability. Furthermore, we did not have information on obstetric or neonatal interventions that could influence risks. However, pregnancies with T1D are managed according to national recommendations, which include supervision regarding risks of fetal hypoxia. Our HbA1c data were limited to measures –365 to +90 days from conception. Ideally, analyses should include measures at conception and during organogenesis (first 8 weeks of pregnancy). In Sweden, women with T1D are transferred from the diabetes clinic to specialist antenatal care when pregnancy is recognized and measures of HbA1c in pregnancy are often not registered in the NDR. We lacked information on other potential risk factors for ASD, such as maternal medications, drug abuse and exposure to pollutants.^8^ However, we have no reason to believe that these exposures would differ between mothers with and without T1D. The rare exposure, T1D, and outcome did not allow a reliable adjustment for familial confounding,^43^ and we could not reliably determine the date of onset of T1D but we believe maternal age is a fair approximation. Nevertheless, it is possible that the observed association between T1D and ASD is partly caused by residual confounding.

The mechanism underlying the association between T1D and ASD is unclear. Some studies support that hyperglycaemia during fetal life may lead to permanent changes in neuronal networks.^44^ Hyperglycaemia induces inflammation and oxidative stress,^45^ which in turn have been identified as potential risk factors for ASD.^8^ Still, our analyses do not support any additional risk with higher HbA1c once T1D diagnosis has been adjusted for.

## Conclusion

T1D in pregnancy was associated with increased risks of ASD in the offspring. Twenty percent of the total effect was accounted for by the pathway through pre-term birth. Higher HbA1c was not associated with higher ASD risk, beyond the association explained by the T1D diagnosis itself.

A higher awareness for autism risk in offspring to mothers with T1D may be warranted, especially considering the additional effect of pre-term birth.

## Ethics approval

This study was conducted according to the Helsinki Declaration. The study was approved by the national Swedish ethics review board, Sweden (2017/1875–31/2; 2018/1864–32). No individual-level consent was required and all data used were anonymized.

## Supplementary Material

dyac116_Supplementary_Data

## Data Availability

Data cannot be shared publicly owing to restrictions by law. Data are available from the National Medical Registries in Sweden after approval by the Swedish Ethical Review Authority. The Swedish Birth Register ’s URL is https://www.socialstyrelsen.se/statistik-och-data/bestalla-data-och-statistik and for the Swedish Ethical Review Authority it is https://etikprovningsmyndigheten.se.

## References

[1] McCance DR. Pregnancy and diabetes. Best Pract Res Clin Endocrinol Metab 2011;25:945–58. 22115168 10.1016/j.beem.2011.07.009

[2] Persson M , Norman M, Hanson U. Obstetric and perinatal outcomes in type 1 diabetic pregnancies: a large, population-based study. Diabetes Care 2009;32:2005–2009. 19675195 10.2337/dc09-0656 PMC2768194

[3] Buxbaum JD , Hof PR, The Neuroscience of Autism Spectrum Disorders. Academic Press, 2013. http://www.sciencedirect.com/science/book/9780123919243 (12 September 2013, date last accessed).

[4] Baxter AJ , Brugha TS, Erskine HE, Scheurer RW, Vos T, Scott JG. The epidemiology and global burden of autism spectrum disorders. Psychol Med 2015;45:601–13. 25108395 10.1017/S003329171400172X

[5] Elsabbagh M , Divan G, Koh YJ et al Global prevalence of autism and other pervasive developmental disorders. Autism Res 2012;5:160–79. 22495912 10.1002/aur.239 PMC3763210

[6] Sandin S , Lichtenstein P, Kuja-Halkola R, Hultman C, Larsson H, Reichenberg A. The heritability of autism spectrum disorder. JAMA 2017;318:1182–84. 28973605 10.1001/jama.2017.12141 PMC5818813

[7] Bai D , Yip BHK, Windham GC et al Association of genetic and environmental factors with autism in a 5-country cohort. JAMA Psychiatry 2019;76:1035. 31314057 10.1001/jamapsychiatry.2019.1411 PMC6646998

[8] Modabbernia A , Velthorst E, Reichenberg A. Environmental risk factors for autism: an evidence-based review of systematic reviews and meta-analyses. Mol Autism 2017;8:13. 28331572 10.1186/s13229-017-0121-4 PMC5356236

[9] Cordero C , Windham GC, Schieve LA et al Maternal diabetes and hypertensive disorders in association with autism spectrum disorder. Autism Res 2019;12:967–75. 30969030 10.1002/aur.2105 PMC6546522

[10] Li M , Fallin MD, Riley A et al The association of maternal obesity and diabetes with autism and other developmental disabilities. Pediatrics 2016;137:e20152206. 26826214 10.1542/peds.2015-2206 PMC4732357

[11] Lyall K , Schmidt RJ, Hertz-Picciotto I. Maternal lifestyle and environmental risk factors for autism spectrum disorders. Int J Epidemiol 2014;43:443–64. 24518932 10.1093/ije/dyt282 PMC3997376

[12] Krakowiak P , Walker CK, Tancredi D et al Autism-specific maternal anti-fetal brain autoantibodies are associated with metabolic conditions. Autism Res 2017;10:89–98. 27312731 10.1002/aur.1657 PMC5164872

[13] Nahum Sacks K , Friger M, Shoham-Vardi I et al Prenatal exposure to gestational diabetes mellitus as an independent risk factor for long-term neuropsychiatric morbidity of the offspring. Am J Obstet Gynecol 2016;215:380.e1–e7. 10.1016/j.ajog.2016.03.030 27018463

[14] Xiang AH , Wang X, Martinez MP et al Association of maternal diabetes with autism in offspring. JAMA 2015;313:1425–34. 25871668 10.1001/jama.2015.2707

[15] Ornoy A , Weinstein-Fudim L, Ergaz Z. Genetic syndromes, maternal diseases and antenatal factors associated with autism spectrum disorders (ASD). Front Neurosci 2016;10:316. 27458336 10.3389/fnins.2016.00316 PMC4933715

[16] Jo H , Eckel SP, Chen JC et al Gestational diabetes mellitus, prenatal air pollution exposure, and autism spectrum disorder. Environ Int 2019;133: 105110. 31610366 10.1016/j.envint.2019.105110 PMC7250244

[17] Xu G , Jing J, Bowers K, Liu B, Bao W. Maternal diabetes and the risk of autism spectrum disorders in the offspring: a systematic review and meta-analysis. J Autism Dev Disord 2014;44:766–75. 24057131 10.1007/s10803-013-1928-2 PMC4181720

[18] Wan H , Zhang C, Li H, Luan S, Liu C. Association of maternal diabetes with autism spectrum disorders in offspring: a systemic review and meta-analysis. Medicine (Baltimore) 2018;97:e9438. 29480832 10.1097/MD.0000000000009438 PMC5943853

[19] Xiang AH , Chow T, Martinez MP et al Hemoglobin A1c levels during pregnancy and risk of autism spectrum disorders in offspring. JAMA 2019;322:460. 31177273 10.1001/jama.2019.8584 PMC6563556

[20] Xiang AH , Wang X, Martinez MP, Page K, Buchanan TA, Feldman RK. Maternal type 1 diabetes and risk of autism in offspring. JAMA 2018;320:89–91. 29936530 10.1001/jama.2018.7614 PMC6134431

[21] Ludvigsson JF , Andersson E, Ekbom A et al External review and validation of the Swedish national inpatient register. BMC Public Health 2011;11:450. 21658213 10.1186/1471-2458-11-450 PMC3142234

[22] Mayer-Davis EJ , Kahkoska AR, Jefferies C et al ISPAD Clinical Practice Consensus Guidelines 2018: definition, epidemiology, and classification of diabetes in children and adolescents. Pediatr Diabetes 2018;19(Suppl 27):7–19. 10.1111/pedi.12773 PMC7521365 30226024

[23] Classification and Diagnosis of Diabetes: Standards of Medical Care in Diabetes—2019. Diabetes Care 2019;42(Suppl 1):S13–28. 30559228 10.2337/dc19-S002

[24] American Diabetes Association. 13. Management of diabetes in pregnancy: standards of medical care in diabetes—2018. Diabetes Care 2018;41(Suppl 1):S137–43. 29222384 10.2337/dc18-S013

[25] Gudbjörnsdottir S , Cederholm J, Nilsson PM, Eliasson BRN; Steering Committee of the Swedish National Diabetes Register. The National Diabetes Register in Sweden: an implementation of the St. Vincent Declaration for Quality Improvement in Diabetes Care. Diabetes Care 2003;26:1270–76. 12663609 10.2337/diacare.26.4.1270

[26] Henderson JJ , McWilliam OA, Newnham JP, Pennell CE. Preterm birth aetiology 2004-2008. Maternal factors associated with three phenotypes: spontaneous preterm labour, preterm pre-labour rupture of membranes and medically indicated preterm birth. J Matern Fetal Neonatal Med 2012;25:642–47. 21827362 10.3109/14767058.2011.597899

[27] Wu S , Wu F, Ding Y, Hou J, Bi J, Zhang Z. Advanced parental age and autism risk in children: a systematic review and meta-analysis. Acta Psychiatr Scand 2017;135:29–41. 27858958 10.1111/acps.12666

[28] Body mass index—BMI. https://www.euro.who.int/en/health-topics/disease-prevention/nutrition/a-healthy-lifestyle/body-mass-index-bmi (11 February 2021, date last accessed).

[29] Loef B , Meulman I, Herber G-CM et al Socioeconomic differences in healthcare expenditure and utilization in the Netherlands. BMC Health Serv Res 2021;21:643. 34217287 10.1186/s12913-021-06694-9 PMC8254290

[30] Butwicka A , Frisén L, Almqvist C, Zethelius B, Lichtenstein P. Risks of psychiatric disorders and suicide attempts in children and adolescents with type 1 diabetes: a population-based cohort study. Diabetes Care 2015;38:453–59. 25650362 10.2337/dc14-0262 PMC4338504

[31] Hossain MM , Khan N, Sultana A et al Prevalence of comorbid psychiatric disorders among people with autism spectrum disorder: an umbrella review of systematic reviews and meta-analyses. Psychiatry Res 2020;287:112922. 32203749 10.1016/j.psychres.2020.112922

[32] Agrawal S , Rao SC, Bulsara MK, Patole SK. Prevalence of autism spectrum disorder in preterm infants: a meta-analysis. Pediatrics 2018;142:e20180134. 30076190 10.1542/peds.2018-0134

[33] Ronald A , Hoekstra RA. Autism spectrum disorders and autistic traits: a decade of new twin studies. Am J Med Genet B Neuropsychiatr Genet 2011;156B:255–74. 21438136 10.1002/ajmg.b.31159

[34] VanderWeele TJ. A unification of mediation and interaction: a four-way decomposition. Epidemiology 2014;25:749–61. 25000145 10.1097/EDE.0000000000000121 PMC4220271

[35] VanderWeele T. Explanation in Causal Inference: Methods for Mediation and Interaction. New York: Oxford University Press, 2015.

[36] Smith LH , VanderWeele TJ. Mediational E-values: approximate sensitivity analysis for unmeasured mediator–outcome confounding. Epidemiology 2019;30:835–37. 10.1097/EDE.0000000000001064 31348008 PMC6768718

[37] Efron B , Tibshirani RJ. An Introduction to the Bootstrap, 1st edn. New York: Chapman and Hall/CRC, 1994.

[38] Raile K , Galler A, Hofer S et al Diabetic nephropathy in 27,805 children, adolescents, and adults with type 1 diabetes: effect of diabetes duration, A1C, hypertension, dyslipidemia, diabetes onset, and sex. Diabetes Care 2007;30:2523–28. 17630266 10.2337/dc07-0282

[39] Januszewski AS , Sutanto SS, McLennan S et al Shorter telomeres in adults with Type 1 diabetes correlate with diabetes duration, but only weakly with vascular function and risk factors. Diabetes Res Clin Pract 2016;117:4–11. 27329016 10.1016/j.diabres.2016.04.040

[40] Grambsch PM , Therneau TM. Proportional hazards tests and diagnostics based on weighted residuals. Biometrika 1994;81:515–26.

[41] Morahan G , Mehta M, James I et al; Type 1 Diabetes Genetics Consortium. Tests for genetic interactions in type 1 diabetes: linkage and stratification analyses of 4,422 affected sib-pairs. Diabetes 2011;60:1030–40. 21266329 10.2337/db10-1195 PMC3046821

[42] Chaste P , Leboyer M. Autism risk factors: genes, environment, and gene-environment interactions. Dialogues Clin Neurosci 2012;14:281–92. 23226953 10.31887/DCNS.2012.14.3/pchaste PMC3513682

[43] Frisell T , Öberg S, Kuja-Halkola R, Sjölander A. Sibling comparison designs: bias from non-shared confounders and measurement error. Epidemiology 2012;23:713–20. 22781362 10.1097/EDE.0b013e31825fa230

[44] Plagemann A. Maternal diabetes and perinatal programming. Early Hum Dev 2011;87:743–47. 21945359 10.1016/j.earlhumdev.2011.08.018

[45] van Niekerk G , Christowitz C, Conradie D, Engelbrecht AM. Insulin as an immunomodulatory hormone. Cytokine Growth Factor Rev 2020;52:34–44. 31831339 10.1016/j.cytogfr.2019.11.006

