# Supplementary material for: Maternal type 1 diabetes, pre-term birth and risk of autism spectrum disorder–a prospective cohort study
Source: Int J Epidemiol. 2022 Jun 3;52(2):377–85. doi: 10.1093/ije/dyac116 (PMC10114059; doi:10.1093/ije/dyac116)
Supplement: dyac116_Supplementary_Data [file dyac116_supplementary_data.pdf]

Table	Content	Page
Table S1	Diagnostic codes	3
Table S2	Cohort description for mothers with type-1 diabetes (T1D), with and without HbA1c data in the register	4
Table S3	Hazard ratios for ASD in offspring of mothers with type-1 diabetes compared with mothers without type-1 diabetes in subgroups by maternal body mass index (BMI kg/m^2)	7
Table S4	Hazard ratios for ASD in offspring of mothers with type-1 diabetes compared with mothers without type-1 diabetes in subgroups of size for gestational age	8
Table S5	Hazard ratios for ASD in two time periods : children born 1998-2002 and 2003-2007	9
Table S6	Hazard ratios for ASD in subgroups of male and female offspring	10
Table S7	Relative risk (Hazard ratios) of ASD in offspring of mothers with type-1 diabetes compared to mother without type-1 diabetes – by sex and preterm/term births	11
Table S8	Hazard ratios for Autistic Disorder in offspring of mothers with type-1 diabetes and mediation by preterm birth	12
Table S9	Mediation of effect from maternal T1D to offspring ASD, mediated by preterm birth with interaction between exposure and mediator	14
Table S10	SAS code, proc causalmed, for the mediation analyses	17

Figure	Content	Page
Figure S1	Inverse Kaplan-Meier Survival curves estimating age-cumulative probability of ASD in offspring of mothers with type-1 diabetes (red) and without type-1 diabetes (blue)	18
Figure S2	Inverse Kaplan-Meier Survival curves estimating age-cumulative probability of ASD in offspring of mothers with type-1 diabetes in term (blue) and preterm (red) born children	19
Figure S3	Examining proportional hazards assumption for T1D effect using standardized Schoenfeld residuals	20
Figure S4	Inverse Kaplan-Meier Survival curves estimating age-cumulative probability of ASD in offspring of mothers with type-1 diabetes in male (blue) and female (red) offspring	21
Figure S5	Hazard ratio of ASD and two-sided 95% confidence intervals associated with advancing maternal age (x-axis) in offspring of mothers with T1D (red) compared to mothers without T1D (blue)	22
Figure S6	Ascertainment of HbA1c	23
Figure S7	Directed acyclic graph (DAG) depicting the mediation analysis	24

Table S1. Diagnostic codes

**Diagnosis**	**ICD-10 code**
Autistic Disorder	F84.0 (Childhood Autism)
Other Autism Spectrum Disorder	F84.9 Pervasive developmental disorder, unspecified<br>F84.1 Atypical Autism<br>F84.8 Other pervasive developmental disorders<br>F84.5 Asperger's Syndrome
Type 1 diabetes	E 10

ICD: International Classification of Diseases

Table S2. Cohort description for mothers with type-1 diabetes (T1D), with and without HbA1c data in the register

**Covariate**	**Mothers with HbA1c Number of children (%)**	**Mothers without HbA1c Number of children (%)**
Children (% male)	4,945 (50.8%)	3,058 (51.3%)
Autistic Spectrum Disorder (ASD)	103 (2.1%)	93 (3.0%)
Incidence rate of ASD per 100,000 person years (total follow up)	279 (36,946)	272 (34,201)
Autistic Disorder (AD)	58 (1.17%)	45 (1.5%)
Incidence rate of AD per 100,000 person years (total follow up)	156 (37,136)	131 (34,398)
Preterms (<37 weeks gestation)	1,182 (23.9%)	677 (22.1%)
Birth year		
1998-2003	711 (14.4%)	1,318 (43.1%)
2004-2009	1,722 (34.8%)	1,049 (34.3%)
2010-2015	2,512 (50.8%)	691 (22.6%)

Table S2. (Continued)

Covariate	Mothers with HbA1c Number of children (%)	Mothers without HbA1c Number of children (%)
Mothers age at delivery		
<20	11 (0.2%)	62 (2.0%)
20-29	1,916 (38.7%)	1,250 (40.9%)
…30-39	2,799 (56.6%)	1,603 (52.4%)
…>=40	219 (4.4%)	143 (4.7%)
Fathers age at delivery		
<20	2 (0.04%)	16 (0.5%)
20-29	1,245 (25.2%)	834 (27.3%)
…30-39	3,038 (61.4%)	1,793 (58.6%)
…>=40	660 (13.3%)	415 (13.6%)
Size for gestational age		
SGA	36 (0.8%)	56 (1.9%)
AGA	2,812 (58.6%)	1,959 (65.8%)
LGA	1,947 (40.6%)	961 (32.3%)
Maternal psychiatric history	886 (17.9%)	460 (15.0%)
Paternal psychiatric history	383 (7.7%)	205 (6.7%)
Mother's BMI (Q1/Median/Q3) *	25.1 (22.9 – 28.1)	25.0 (22.8 – 28.4)
Underweight#	13 (0.3%)	22 (0.8%)
Normal weight#	2,235 (48.7%)	1,302 (49.6%)

Table S2. (Continued)

Covariate	Mothers with HbA1c Number of children (%)	Mothers without HbA1c Number of children (%)
Overweight#	1,596 (34.7%)	814 (31.0%)
Obese#	750 (16.3%)	487 (18.6%)
Maternal education		
Primary	371 (7.5%)	413 (13.5%)
Secondary	2,193 (44.3%)	1,523 (49.8%)
University	2,381 (48.1%)	1,122 (36.7%)
Paternal education		
Primary	463 (9.4%)	429 (14.0%)
Secondary	2,731 (55.2%)	1,675 (54.8%)
University	1,751 (35.4%)	954 (31.2%)

SGA: Small for gestational age; AGA: Appropriate for gestational age; LGA: Large for gestational age; HbA1c: glycated haemoglobin;
Underweight: BMI<18.5, Normal weight: BMI 18.5-25, Overweight: BMI 25-30, Obese: BMI≥30
* Q1: 1st quartile (25th percentile), Q3: 3rd quartile (75th percentile)

Table S3 Hazard ratios for ASD in offspring of mothers with type-1 diabetes compared with mothers without type-1 diabetes in subgroups by maternal body mass index (BMI kg/m^2)

**Subgroup**	**Number of subjects**	**Rate (Cases; Person years)**	**Crude hazard ratio (95% CI)**	**Adjusted hazard ratio (95% CI)**
Normal weight BMI -25	815,881	157 (12,154; 7,747,079)	1.61 (1.28 – 2.01)	1.47 (1.18 – 1.84)
Overweight BMI 25-30	307,980	196 (5,704; 2,906,995)	1.34 (1.02 – 1.76)	1.26 (0.96 – 1.66)
Obesity BMI 30-	145,268	286 (3,735; 1,305,037)	1.19 (0.85 – 1.67)	1.11 (0.79 – 1.55)

Model adjusted for birth year and parental age by natural splines, parental psychiatric history and education; ASD: Autism Spectrum Disorder; CI: Two-sided 95% confidence interval; Rate: Cases per 100,000 person years

Table S4 Hazard ratios for ASD in offspring of mothers with type-1 diabetes compared with mothers without type-1 diabetes in subgroups of size for gestational age

**Subgroup**	**Number of subjects**	**Rate (Cases; Person years)**	**Crude HR (95% CI)**	**Adjusted HR (95% CI)**
SGA	25,909	344 (859; 249,604)	1.66 (0.69 – 4.00)	1.57 (0.65 – 3.78)
AGA	1,283,045	180 (22,131; 12,292,766)	1.53 (1.28 – 1.84)	1.40 (1.17 – 1.68)
LGA	53,775	213 (1,120; 527,040)	1.41 (1.10 – 1.79)	1.29 (1.01 – 1.65)

Rate: Cases per 100,000 person years. SGA: Small for gestational age; AGA: Appropriate for gestational age; LGA: Large for gestational age; CI: Two-sided 95% confidence interval; ASD: Autism Spectrum Disorder;
Crude HR: Cox regression adjusted for birth year and parental age by natural cubic splines;
Adjusted HR: Cox regression adjusted for birth year and parental age by natural cubic splines and parental psychiatric history and education;

Table S5 Hazard ratios for ASD in offspring of mothers with type-1 diabetes compared with mothers without type-1 diabetes in children born 1998-2002 and 2003-2007. All children followed for 10 years to allow a fair comparison of the two time periods.

**Model**	**Number of subjects**	**Rate (Cases; Person years)**	**Hazard ratio (95% Confidence interval)**
Children born 1998-2002#	356,461	93 (3,277; 3,526,705)	1.20 (0.78 – 1.85)
Children born 2003-2007#	397,292	158 (6,152; 3,883,824)	1.26 (0.96 – 1.66)

#Adjusting for birth years by natural cubic splines, maternal and paternal age by natural cubic splines, parental psychiatric history (yes/no) and maternal and paternal education at delivery

Table S6. Hazard ratios for ASD in subgroups of male and female offspring

Model	Males			Females		
	**Number of subjects**	**Rate (Cases; Person years)**	**Hazard ratio (95% CI)**	**Number of subjects**	**Rate (Cases; Person years)**	**Hazard ratio (95% CI)**
Crude	723,770	256 (17,738; 6,929,933)	1.42 (1.20 – 1.69)	682,880	110 (7,203; 6,577,914)	1.81 (1.42 – 2.31)
Adjusted#			1.32 (1.11 – 1.56)			1.63 (1.28 – 2.08)

adjusted for birth year and parental age by natural spline and parental psychiatric history and education. ASD: Autism Spectrum Disorder; CI: Two-sided 95% confidence interval; Rate: Cases per 100,000 person years

Table S7 Relative risk (Hazard ratios) of ASD in offspring of mothers with type-1 diabetes compared to mother without type-1 diabetes – by sex and preterms/term births.

Subgroup by sex	Preterm born children			Term born children		
	Number of subjects	Rate (Cases; Person years)	Hazard ratio (95% CI)	Number of subjects	Rate (Cases; Person years)	Hazard ratio (95% CI)
Males	43,948	352 (1,497; 424,737)	1.05 (0.75 – 1.47)	679,822	250 (16,241; 6,505,197)	1.32 (1.08 – 1.62)
Girls	37,967	165 (612; 371,763)	1.58 (1.03 – 2.43)	644,913	106 (6,591; 6,206,151)	1.48 (1.10 – 1.99)

Note: Adjusted models, adjusted for birth year and parental age by natural spline and parental psychiatric history and education; ASD: Autism Spectrum Disorder; CI: Two-sided 95% confidence interval; Rate: Cases per 100,000 person years

Table S8 Hazard ratios for Autistic Disorder (AD) in offspring of mothers with type-1 diabetes and mediation of effect from maternal T1D to offspring ASD, mediated by preterm birth

Mediation analyses assessing risk by relative risks (RR) from log-binomial regression T1D → AD and for preterm → AD.

Model	Number of subjects	AD (%)	Direct effect£ RR (95% CI)	E-value (95% CI)	Mediation effect£ RR (95% CI)	E-value (95% CI)	Proportion Mediation£ % (95% CI)	Total Effect RR (95% CI)
Crude#	1,406,650	13,380 (0.95%)	1.30 (1.06-1.55)	1.93 (1.32-2.47)	1.10 (1.08-1.12)	1.44 (1.39-1.49)	30 (21-59)	1.44 (1.16-1.70)
Adj##	1,406,650	13,380 (0.95%)	1.19 (0.98-1.46)	1.68 (1.00-2.28)	1.09 (1.07-1.10)	1.39 (1.35-1.43)	35 (20-124)	1.30 (1.06-1.59)
Single-tons##	1,365,518	12,943 (0.95%)	1.19 (0.97-1.43)	1.66 (1.00-2.22)	1.10 (1.08-1.11)	1.42 (1.37-1.47)	38 (23-124)	1.30 (1.07-1.57)
Males##	723,770	9,731 (1.34%)	1.10 (0.86-1.39)	1.43 (1.00-2.12)	1.08 (1.06-1.10)	1.37 (1.32-1.43)	47 (-138-910)	1.19 (0.93-1.49)
Females	682,880	3,649	1.50 (1.06-2.04)	2.36 (1.32-3.50)	1.09 (1.06-1.12)	1.40 (1.31-1.49)	21 (12-59)	1.63 (1.14-2.23)

##		(0.53%)						

ASD: Autism Spectrum Disorder; AD: Autistic Disorder; RR: Relative Risk from log-binomial regression; CI: Two-sided confidence interval from bootstrapping 1,000 samples. #Outcome and mediation model adjusted for birth year (1998-2002, 2003-2007, 2008-2012, 2013-2015)

##Outcome and mediation model additionally adjusted for maternal age (<20, 20-24, 24-29, 30-34, 34-39, 40-44, >45), paternal age (<20, 20-24, 24-29, 30-34, 34-39, 40-44, >45), maternal psychiatric history at delivery (yes/no), paternal psychiatric history at delivery (yes/no), Maternal education attainment at delivery (Grundskola, Gymnasium, University) and Paternal education attainment at delivery (Grundskola, Gymnasium, University).

£: Direct effect often referred to as "Controlled Direct Effect" and Mediation usually referred to as "Natural Indirect Effect (NIE)"

Note: "Controlled Direct Effect" is the RR of ASD comparing offspring to mothers with T1D diagnosis to offspring to mothers without T1D diagnosis, when the preterm covariates are assigned the same value, e.g. term. "Natural Indirect Effect" is the RR of ASD comparing offspring born preterm to offspring born term assuming all are born to mothers diagnosed with T1D. "Total Effect" is the RR of ASD comparing preterm born offspring to mothers with T1D diagnosis to term born offspring to mothers without T1D diagnosis, i.e. the RR comparing assumed highest risk group to lowest risk group.

Table S9. Mediation of effect from maternal T1D to offspring ASD, mediated by preterm birth with interaction between exposure and mediator

Note: The results below complement the results from the model without interaction in manuscript Table 4 where the total effect was divided in 'natural direct effect' (NDE) and 'natural indirect effect' (NIE). When introducing an interaction term in the model the NIE can be sub-divided into 'pure indect effect' (PIE), for the mediation without influence if potential interaction, and a additional 'indirect mediated interaction' (IMD). There was no support in the data for IMD and interaction.

Mediation analyses assessing risk by relative risks (RR) from log-binomial regression T1D → ASD and for preterm → ASD.

Effect estimates in the mediation analyses	Full dataset 1,406,650 subjects; 24,941 ASD (1.77%)		Subset: Male offspring		Subset: Female offspring	
	**Crude model RR (95% CI)**	**Adjusted model RR (95% CI)**	**Crude model RR (95% CI)**	**Adjusted model RR (95% CI)**	**Crude model RR (95% CI)**	**Adjusted model RR (95% CI)**
**Total effect**	1.51 (1.29 – 1.73)	1.38 (1.19 – 1.58)	1.41 (1.17 – 1.65)	1.30 (1.09 – 1.52)	1.80 (1.40 – 2.22)	1.60 (1.23 – 2.02)

E-value	2.39 (1.91 – 2.86)	2.10 (1.67 – 2.54)	2.16 (1.62 – 2.69)	1.93 (1.40 – 2.41)	2.99 (2.15 – 3.87)	2.59 (1.75 – 3.46)
**NDE**	1.43 (1.21 – 1.67)	1.33 (1.14 – 1.56)	1.37 (1.13 – 1.63)	1.29 (1.07 – 1.54)	1.61 (1.21 – 2.03)	1.47 (1.08 – 1.87)
proportion	84 (62 – 102)	87 (55 – 107)	91 (59 – 120)	97 (56 – 132)	77 (45 – 100)	78 (32 – 105)
E-value	2.21 (1.73 – 2.72)	1.99 (1.54 – 2.49)	2.08 (1.51 – 2.65)	1.90 (1.34 – 2.45)	2.60 (1.71 – 3.48)	2.30 (1.39 – 3.15)
**PIE**#	1.08 (1.07 – 1.09)	1.07 (1.06 – 1.08)	1.07 (1.06 – 1.08)	1.06 (1.05 – 1.07)	1.08 (1.06 – 1.11)	1.07 (1.05 – 1.09)
proportion	15 (10 – 26)	17 (11 – 33)	17 (11 – 41)	20 (11 – 65)	11 (6 – 22)	12 (6 – 31)
E-Value	1.37 (1.33 – 1.40)	1.34 (1.30 – 1.37)	1.35 (1.31 – 1.39)	1.31 (1.27 – 1.35)	1.39 (1.32 – 1.45)	1.35 (1.29 – 1.42)
**IMD**#	1.00 (0.92 – 1.10)	0.98 (0.91 – 1.07)	0.97 (0.86 – 1.07)	0.95 (0.87 – 1.04)	1.10 (0.92 – 1.31)	1.07 (0.90 – 1.25)
proportion	1 (-21 – 21)	-4 (-28 – 19)	-8 (-46 – 20)	-16 (-67 – 16)	13 (-12 – 44)	11 (-20 – 48)
E-Value	1.07 (1.00 – 1.43)	1.14 (1.00 – 1.41)	1.22 (1.00 – 1.54)	1.28 (1.00 – 1.57)	1.43 (1.00 – 1.94)	1.33 (1.00 – 1.81)

ASD: Autism Spectrum Disorder; RR: Relative Risk from log-binomial regression; CI: Two-sided confidence interval from bootstrapping 1,000 samples. #Outcome and mediation model adjusted for birth year (1998-2002, 2003-2007, 2008-2012, 2013-2015)

##Outcome and mediation model additionally adjusted for maternal age (<20, 20-24, 24-29, 30-34, 34-39, 40-44, >45), paternal age (<20, 20-24, 24-29, 30-34, 34-39, 40-44, >45), maternal psychiatric history at delivery (yes/no), paternal psychiatric history at delivery (yes/no), Maternal education attainment at delivery ("Grundskola", "Gymnasium", "University") and Paternal education attainment at delivery ("Grundskola", "Gymnasium", "University").

Note: Total effect = NDE + NIE (see table 4 in the manuscript) = NDE + PID + IMD, when interaction term between T1D and preterm birth is included in the model. PIE is the component effect that is due to mediation but not interaction, and IMD is the mediated interaction.

Table S10 SAS code, proc causalmed, for the mediation analyses

```
title1 'Mediation ASD. Crude - without interaction';
proc causalmed descending decomp pall;
   bootstrap bootci(perc bc);
   class t1d(ref=first) prem(ref=first) yearcat(ref='1998-2002');
   model    asd  = t1d prem / link=log dist=binomial;
   mediator prem  = t1d;
   covar    yearcat;
run;

title1 'Mediation ASD. Adjusted - without interaction';
proc causalmed descending decomp pall;
   bootstrap bootci(perc bc);
   class t1d(ref=first) prem(ref=first) yearcat(ref='1998-2002') magecat(ref='25-30') fagecat(ref='25-30')
        mphist(ref=first) fphist(ref=first) meducat(ref='Gymnasium') feducat(ref='Gymnasium');
   model    asd  = t1d prem / link=log dist=binomial;
   mediator prem  = t1d;
   covar    yearcat magecat fagecat mphist fphist meducat feducat;
run;

title1 'Mediation ASD. Adjusted - with interaction';
proc causalmed data=remlib.analysdata descending decomp pall threads=20;
  bootstrap bootci(perc bc);
  class t1d(ref=first) prem(ref=first) yearcat(ref='1998-2002') magecat(ref='25-30') fagecat(ref='25-30')
        mphist(ref=first) fphist(ref=first) meducat(ref='Gymnasium') feducat(ref='Gymnasium');
  model    asd  = t1d | prem / link=log dist=binomial;
  mediator prem  = t1d;
  covar    yearcat magecat fagecat mphist fphist meducat feducat;
run;
```

Figure S1 Inverse Kaplan-Meier Survival curves estimating age-cumulative probability of ASD in offspring of mothers with type-1 diabetes (red) and without type-1 diabetes (blue)

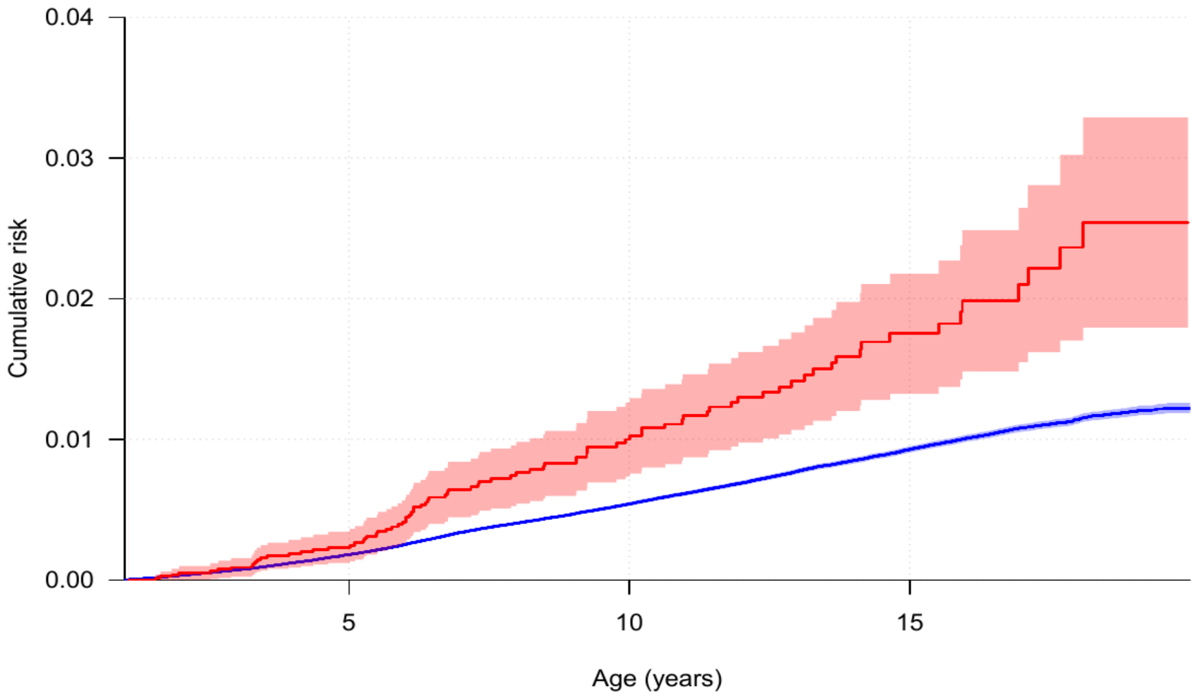

Note: Point estimates and two-sided 95% point-wise confidence intervals

Figure S2 Inverse Kaplan-Meier Survival curves estimating age-cumulative probability of ASD in offspring of mothers with type-1 diabetes in term (blue) and preterm (red) born children

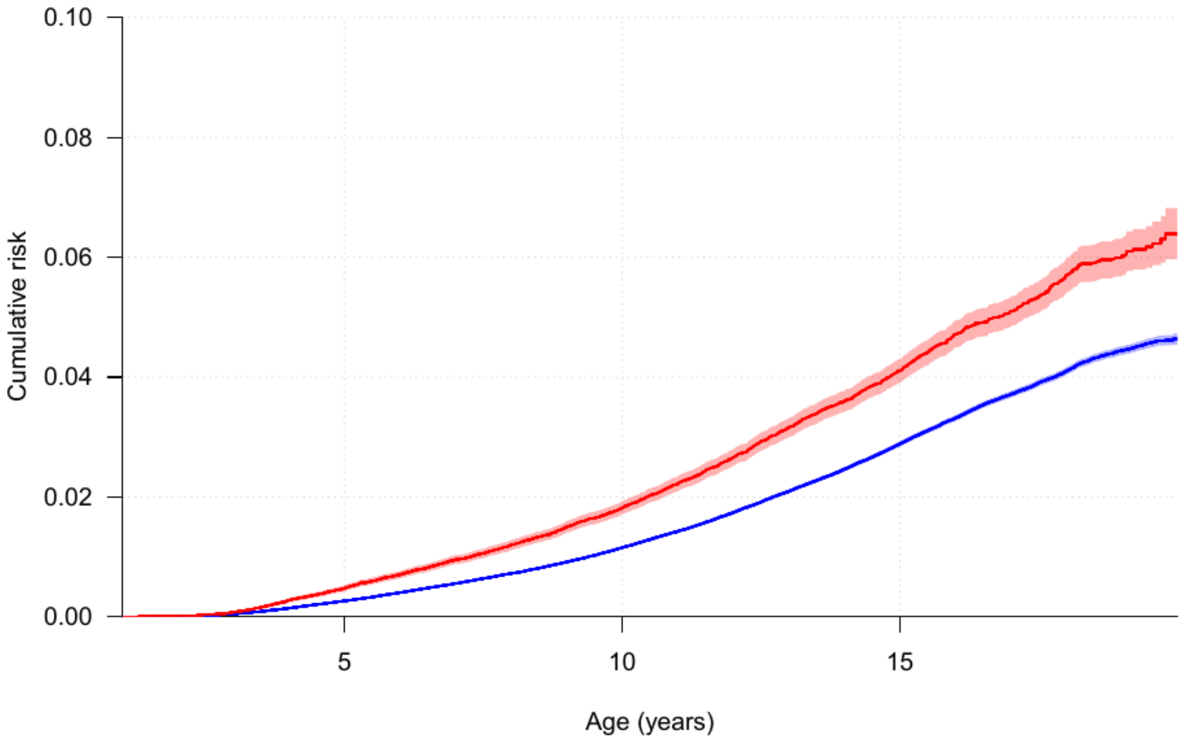

Note: Point estimates and two-sided 95% point-wise confidence intervals

Figure S3 Examining proportional hazards assumption for T1D effect using standardized Schoenfeld residuals

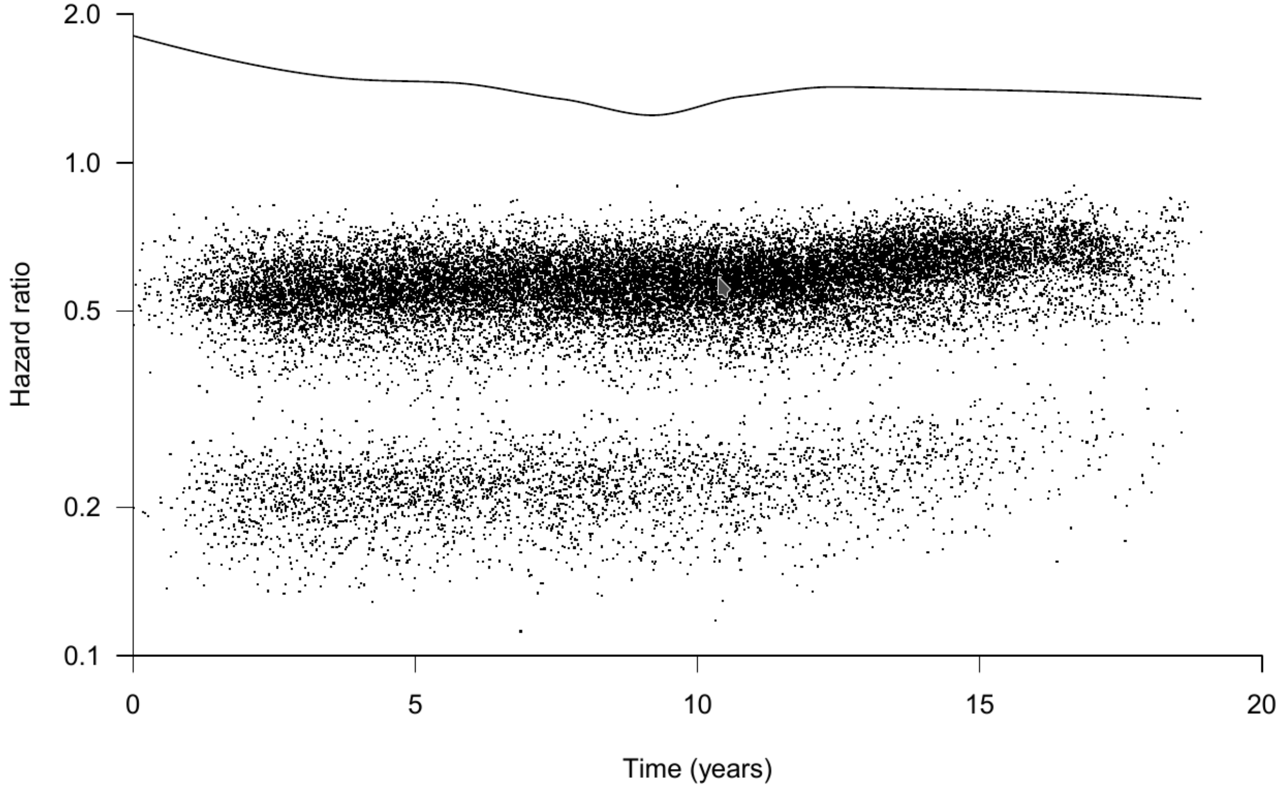

Note: Horizontal line indicate residuals mean at each time (age). The mean estimate any time varying T1D effect.
Note: Using ggcoxzph in the package survminer, https://www.rdocumentation.org/packages/survminer/versions/0.4.8/topics/ggcoxzph

Figure S4 Inverse Kaplan-Meier Survival curves estimating age-cumulative probability of ASD in offspring of mothers with type-1 diabetes in female (blue) and male (red) offspring

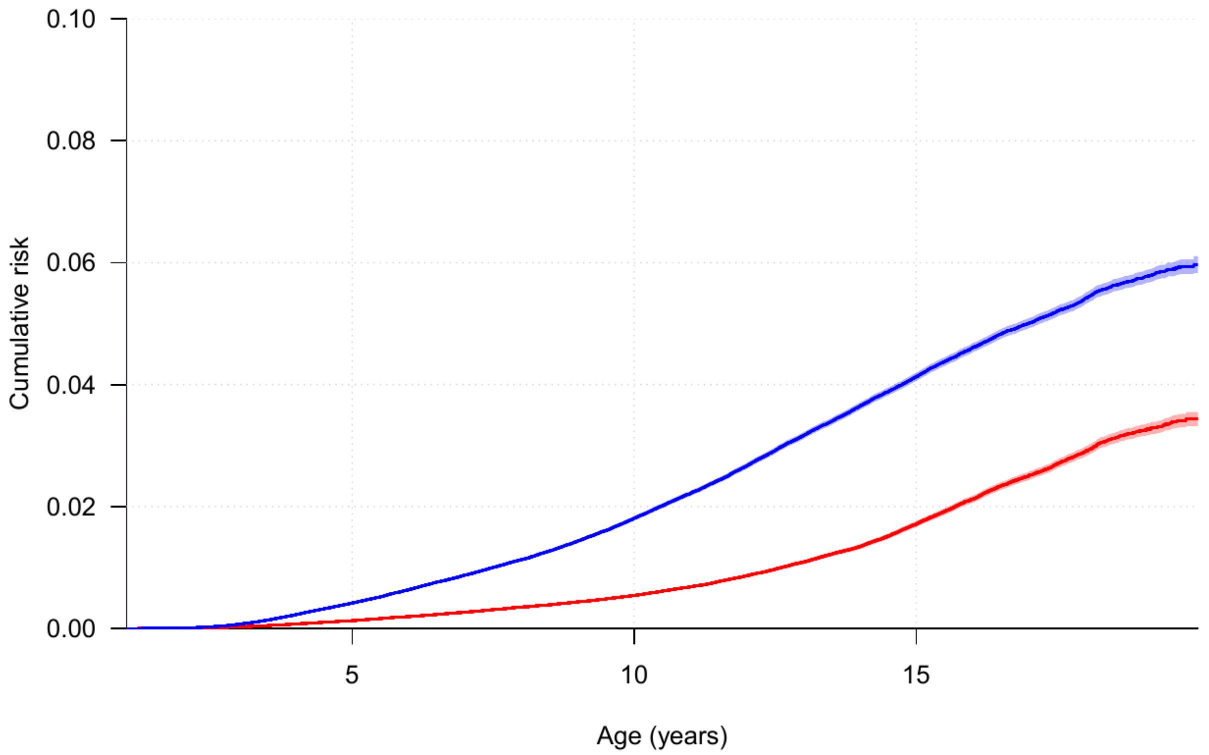

Note: Point estimates and two-sided 95% point-wise confidence intervals

Figure S5 Hazard ratio of ASD and two-sided 95% confidence intervals associated with advancing maternal age (x-axis) in offspring of mothers with T1D (red) compared to mothers without T1D (blue)

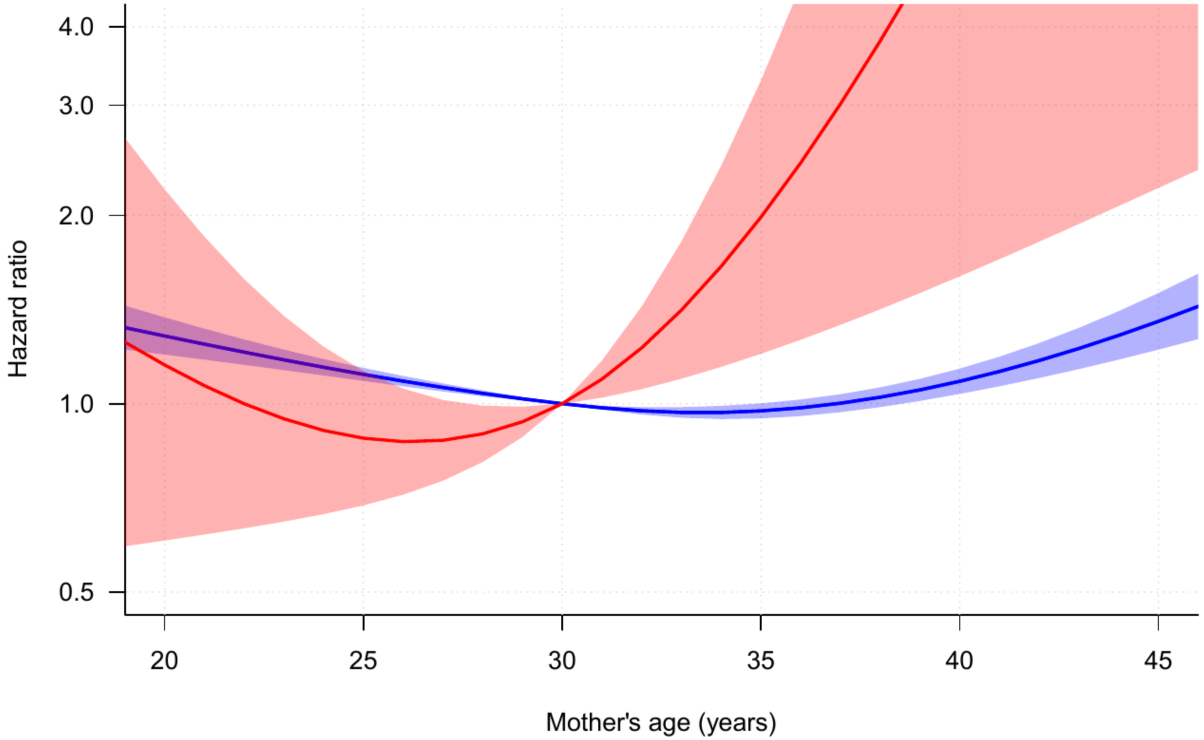

ASD: Autism Spectrum Disorder

Figure S6 Ascertainment of glycated haemoglobin (HbA1c)

In the study 8,003 children were born to 5,049 mothers diagnosed with T1D. We found 4,945 children born to 3,494 mothers diagnosed with T1D, and with at least one HbA1c within –369 days before conception to 90 days after conception in the National Diabetes Register. 103 (2.1%) of these children were diagnosed with ASD. The mean HbA1c was 56 mmol/mol and varied between 34 mmol/mol for the lowest 1% and 102 mmol/mol for the highest 1% of the data distribution. Of the HbA1c data 27% were measured in the interval +-3 months round conception and 45% of the HbA1c sample was ascertained between 3 to 6 months before conception. Half of the HbA1c measure for mothers to children with ASD was estimated taken between -156 and +90 days round conception. Corresponding days for mothers to children without ASD diagnosis was estimate -123 to +90 days round conception.

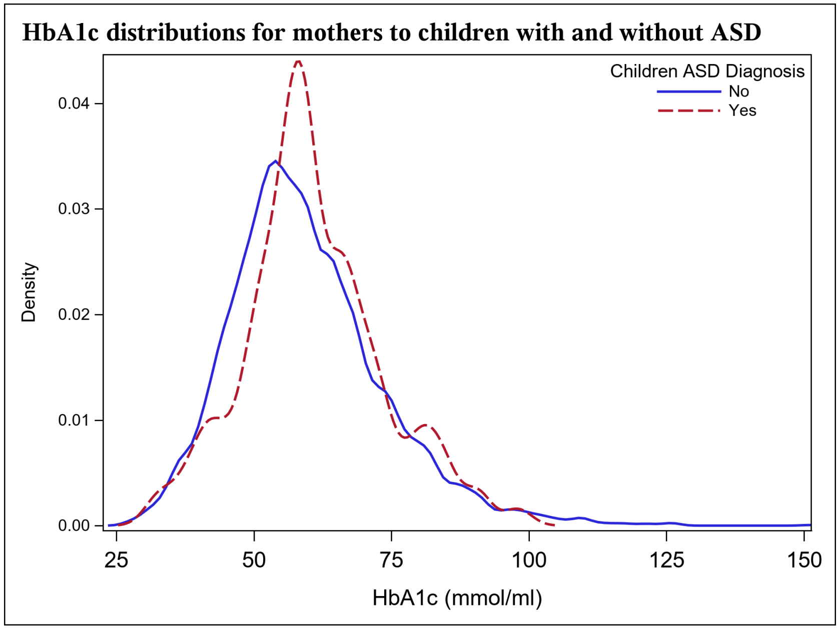

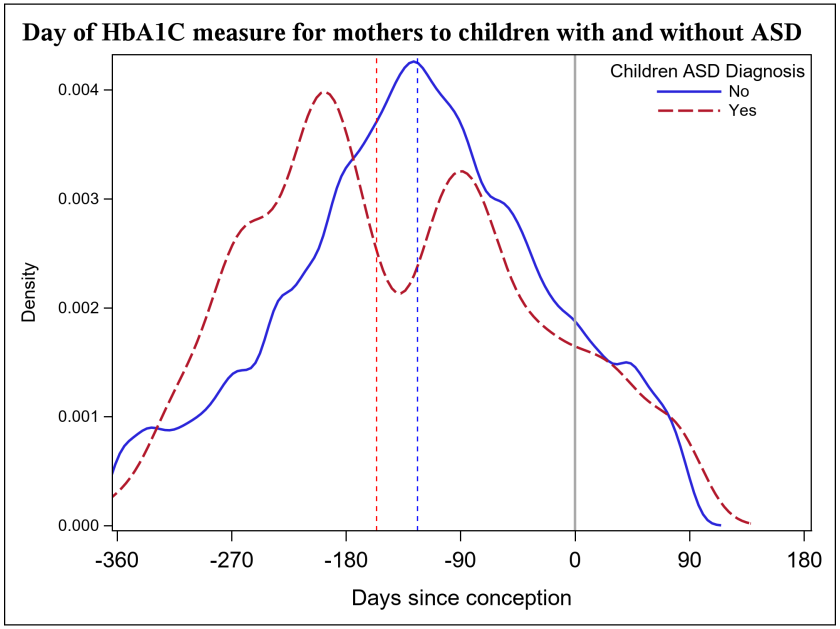

Figure S7 Directed acyclic graph (DAG) depicting the mediation analysis

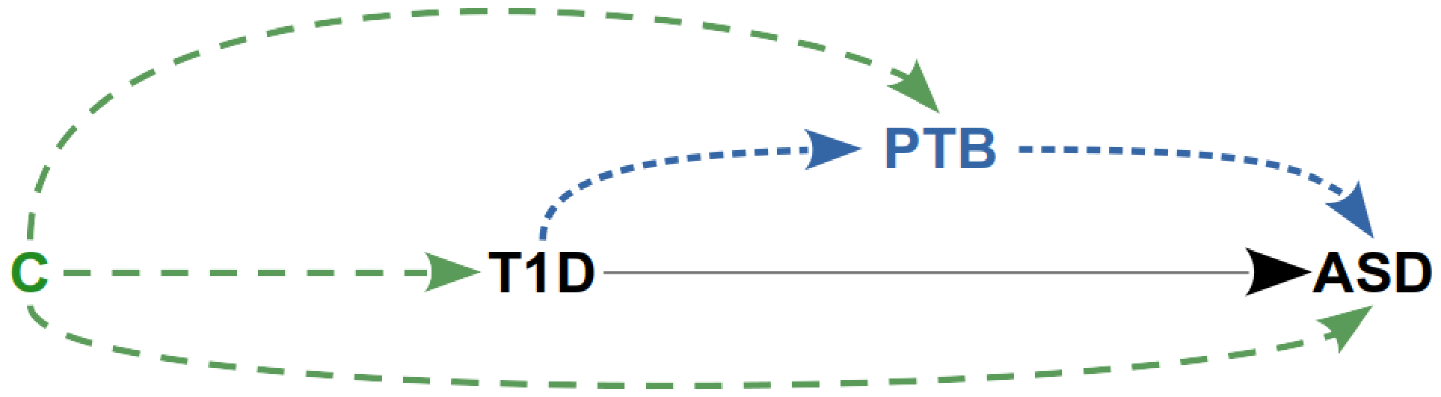

T1D: Maternal T1D diagnosis, diagnosed before offspring birth. **ASD**: Offspring diagnosed with Autism Spectrum Disorder.

PTB: Child born preterm (before 37 weeks of gestation). **C**: Confounding factors in the associations between T1D, PTB and ASD. In our study we adjusted for potential counfounding due to offspring year of birth, maternal and paternal age at the delivery, Presence of maternal and paternal psychiatric history at delivery and maternal and paternal highest attained education at delivery of the child.
